# Effect of Pilates Exercise on Health‐Related Outcomes in Patients With Knee Osteoarthritis: A Systematic Review and Meta‐Analysis

**DOI:** 10.1111/1756-185x.70434

**Published:** 2025-10-09

**Authors:** Túlio Medina Dutra de Oliveira, Diogo Carvalho Felício, José Elias Filho, Rayane Quintão Castro, Fernando Junior da Silva Gomides, Luis Felipe Petronilho Pires, Diogo Simões Fonseca, Carla Malaguti

**Affiliations:** ^1^ Postgraduate Program in Health Faculty of Medicine, Federal University of Juiz de Fora Juiz de Fora Brazil; ^2^ Postgraduate Program in Rehabilitation Sciences and Physical‐Functional Performance Faculty of Physiotherapy, Federal University of Juiz de Fora Juiz de Fora Brazil; ^3^ Faculty of Medicine, Federal University of Juiz de Fora Juiz de Fora Brazil

**Keywords:** exercise, knee osteoarthritis, pain, Pilates, rehabilitation

## Abstract

**Introduction:**

Knee osteoarthritis (KOA) is a chronic, progressive condition characterized by cartilage degeneration, synovial inflammation, and bone changes leading to pain and functional impairment. Despite the availability of various treatment options, including multidisciplinary approaches and muscle strengthening exercises, there remains uncertainty regarding the efficacy of Pilates as a therapeutic intervention for KOA. This highlights the need for a systematic review to synthesize current evidence on the effects of Pilates on health‐related outcomes in this population.

**Objective:**

This systematic review aimed to analyze the effects of Pilates compared to no exercise or conventional exercises on pain in individuals with knee osteoarthritis, as well as on secondary outcomes including function, quality of life, range of motion, balance, and adverse events. A secondary aim is to characterize the key components and implementation characteristics of the Pilates interventions applied in the included studies.

**Methods:**

The review protocol has been registered in PROSPERO under the number CRD42024532727. Searches were conducted in PubMed/MEDLINE, Embase, CINAHL, CENTRAL, Scopus, Web of Science, and SPORTDiscus. Eligible studies included randomized controlled trials (RCTs) that investigated the impact of Pilates exercises in patients aged ≥ 18 years, diagnosed with KOA according to the Kellgren and Lawrence criteria or the American College of Rheumatology. Studies including participants with systemic arthritis, knee joint surgery within the past 12 months, lower extremity arthroplasty, intra‐articular steroid injections within the past 6 months, or any neurological conditions were excluded. The risk of bias was assessed using the RoB 2 tool, and the quality of evidence was evaluated using the GRADE approach. Meta‐analyses used random‐effects models, with standardized mean differences (SMDs) and heterogeneity analyzed using *I* ^2^ statistics.

**Results:**

Eleven studies involving 476 participants were included, of which seven contributed to the quantitative synthesis. Based on this analysis, Pilates exercises reduced pain compared to no intervention (SMD −1.09; 95% CI −2.04 to −0.14; *I* ^2^ = 66%; *p* = 0.02; 3 studies; *n* = 66; low‐quality evidence), but did not demonstrate superiority over conventional exercises (SMD −0.28; 95% CI −1.06 to 0.50; *I* ^2^ = 86%; *p* = 0.49; 5 studies; *n* = 210; very low‐quality evidence). No significant improvement in knee health, assessed by the WOMAC total score, was found when compared to conventional exercises (SMD −0.14; 95% CI −1.12 to 0.85; *I* ^2^ = 91%; *p* = 0.78; 4 studies; *n* = 202; very low‐quality evidence), but knee range of motion increased with Pilates (SMD 1.07; 95% CI 0.56 to 1.57; *I* ^2^ = 0%; *p* = 0.0001; 2 studies; *n* = 70; low‐quality evidence). The qualitative analysis revealed evidence of improvements in balance, proprioception, and quality of life in some individual studies. However, the quality of the evidence was considered very low to low.

**Conclusion:**

The Pilates method may be an effective alternative for the rehabilitation of patients with KOA, particularly in reducing pain compared to no intervention. However, it did not demonstrate superiority over conventional exercises in pain reduction or knee health improvement. Pilates was more effective in increasing range of motion compared to conventional exercises and showed benefits in proprioception, dynamic balance, and quality of life. The heterogeneity among the studies and the low quality of the evidence suggests caution in the interpretation of the results, with new RCTs potentially impacting the findings of this review.

**Summary**

- The Pilates method has shown a significant reduction in pain in patients with knee osteoarthritis (KOA) compared to the absence of intervention. These findings reinforce the potential of Pilates as a complementary therapeutic approach in the management of chronic pain associated with KOA.
- Pilates resulted in a significant increase in knee range of motion compared to conventional exercises and exhibited similar effects on pain reduction and improvement in knee health. Therefore, as a modality that offers lower‐impact exercise options, it may serve as a viable alternative for individuals with mechanical restrictions or difficulty adhering to traditional exercise regimens. Additionally, according to qualitative evidence, Pilates has shown benefits in proprioception and dynamic balance.
- This systematic review employed a robust methodological approach, including the assessment of evidence quality using the GRADE system and quantitative analysis through meta‐analysis. The identification of substantial heterogeneity and the low quality of evidence underscores the need for future randomized clinical trials to strengthen the scientific foundation regarding the efficacy of Pilates in KOA.

## 1 Introduction

Knee osteoarthritis (KOA) is the most common chronic joint disease, characterized by degeneration and loss of cartilage, synovial inflammation, alteration of periarticular bone with osteophyte formation, and subchondral sclerosis [1]. The global prevalence of KOA among individuals aged 40 years and over is estimated to be 22.9%, affecting approximately 654 million people worldwide. Additionally, by 2020, around 86 million adults aged 20 years and over were diagnosed with KOA annually [2]. In the United States, annual direct healthcare costs exceed $185 billion [3]. KOA symptoms include pain, muscle weakness, stiffness, decreased physical function, mobility, range of motion, and quality of life [4, 5]. The management of KOA is a challenge and requires multidisciplinary approaches that combine pharmacological therapy, surgical interventions, and non‐pharmacological strategies [6]. Of these, muscle strengthening is the strategy most recommended according to guidelines, which can be achieved through resistance exercises [7]. In this context, the Pilates method stands out as being a therapeutic option that can influence increased muscle strength [8].

Developed in the early 20th century, Pilates is based on principles such as breathing control, balance, flexibility, proprioception, and strengthening of the powerhouse—a muscular unit that includes the core, abdominal muscles, lumbar muscles, pelvic floor, and hip region [9, 10]. Studies suggest that Pilates exercises may outperform traditional strengthening programs in reducing pain and disability in individuals with KOA [11]. Furthermore, Pilates has been shown to reduce pain and improve quality of life in patients with rheumatological conditions [12, 13]. Such versatility allows routines to be personalized according to the needs of the patients, highlighting the potential of this method for managing KOA [11, 14].

Despite the growing interest in Pilates as a therapeutic intervention for KOA, the available evidence remains fragmented, hindering its practical application in clinical settings. Therefore, the aim of this systematic review is to synthesize the available evidence on the effects of the Pilates method on pain in individuals with knee osteoarthritis, as well as on secondary outcomes including function, quality of life, range of motion, balance, and adverse events. A secondary aim is to characterize the key components and implementation characteristics of the Pilates interventions applied in the included studies.

## 2 Methods

### 2.1 Study Design

This systematic review was prospectively registered with the international prospective register of systematic reviews (PROSPERO) (Registration number: CRD42024532727, December 2024). It was conducted according to the guidance provided in the Cochrane Handbook [15] and reported in line with the preferred reporting items for systematic reviews and meta‐analyses (PRISMA) guidelines [16].

### 2.2 Search Strategy

The search was carried out in November 2024 using the following databases: PubMed/MEDLINE, Embase, CINAHL, CENTRAL, Scopus, Web of Science, and SPORTDiscus. Search strategies were designed based on the specific recommendations of each database, incorporating descriptors and their variations, combined with the Boolean operator “AND.” No limitations were imposed with regard to publication date or language. Furthermore, the reference lists of articles identified through the search process were manually screened to identify additional studies. The full search strategy is provided in the Supporting Information S1.

### 2.3 Eligibility Criteria

Studies including both men and women, aged 18 years or over, diagnosed with unilateral or bilateral KOA, as confirmed by either the Kellgren and Lawrence grading system [17] through radiographic evaluation, the American College of Rheumatology classification [18] or by an orthopedist, were included. This expansion of the diagnostic criteria, relative to the original protocol, was implemented to maximize the inclusion of relevant randomized controlled trials and ensure that all eligible evidence addressing the review objectives was captured. Studies were excluded where the participants had systemic arthritis, knee joint surgery within the past 12 months, lower extremity arthroplasty, intra‐articular steroid injections within the past 6 months, any neurological conditions, as well as studies with mixed populations (e.g., knee osteoarthritis combined with other conditions such as low back pain, neck pain, or osteoporosis), unless data for participants with knee osteoarthritis were reported separately and met the eligibility criteria. The intervention of interest is the Pilates method, a body conditioning technique developed by Joseph Pilates [19], which aims to improve posture, stability, and movement awareness through controlled exercises, emphasizing the isometric contraction of core muscles. Pilates exercises may include both mat‐ and apparatus‐based routines targeting deep stabilizing muscles such as the multifidus and transversus abdominis. Randomized controlled trials were included that compared the effects of Pilates exercise with other exercise methods, or no exercise, including conventional treatments for KOA. Conference abstracts were eligible for inclusion if they met all predefined eligibility criteria and provided sufficient and usable data.

### 2.4 Outcome Measures

The primary outcome for this review was pain. Secondary outcomes included function, quality of life, range of motion, balance, and adverse events. These outcomes were considered eligible for inclusion if they were assessed using validated measurement instruments or standardized clinical scales [15].

### 2.5 Study Selection and Data Extraction

Two authors (TMDO and RQC) exported the studies identified through the search strategy into EndNote X9 software (Thomson Reuters, Philadelphia, USA) for the purpose of removing duplicates. Subsequently, these two authors independently screened the titles and abstracts to assess the potential eligibility of the studies. The full text of selected articles was then reviewed to confirm final inclusion in the review. Any disagreements between reviewers were resolved through discussion or, if necessary, arbitration by a third reviewer (C.M.). Data extraction, including information on author names, year of publication, study design, age, population characteristics, sample size, exercise protocol, duration, main outcomes, and adverse events, was carried out by two reviewers (TMDO and RQC). Specifically, data on adverse events were systematically extracted from the descriptions presented in the Results and Discussion sections, as well as from the study flowchart, to ascertain whether participant withdrawals were attributable to these events. Any discrepancies between reviewers were resolved through consensus or arbitration by a third reviewer (C.M.).

### 2.6 Risk of Bias

The methodological quality of studies was assessed using the Cochrane Risk of Bias 2 (RoB 2) tool for randomized clinical trials. The RoB 2 tool evaluates quality across five key domains: the randomization process, deviations from the intended interventions, missing data, outcome measurement, and the reporting of results. Studies were classified as having a low risk of bias if all domains are rated as “low‐risk,” a moderate risk of bias if one or more domains are marked as presenting “some concerns,” and a high‐risk of bias if any domain is judged to be “high‐risk” [20].

The tool relied on a consensus‐based approach achieved through independent evaluation by two reviewers (T.M.D.O. and J.E.F.). Any disagreements were resolved by consulting a third reviewer (C.M.) for arbitration.

### 2.7 Quality of Evidence

The quality of the evidence included was evaluated using the grading of recommendations, assessment, development and evaluation (GRADE) system [21]. This approach considers five key domains: risk of bias, inconsistency, indirectness, imprecision, and publication bias. For each domain, the reasons for downgrading were as follows: [1] study design: one level of non‐randomized study; [2] risk of bias: one level if the overall assessment presented some concerns; two levels if the overall assessment was high risk of bias; [3] inconsistency: one level if there was minimal overlap in the effect estimates among the studies; two levels if the confidence intervals of the effect estimates did not overlap; [4] indirectness: one level if there was indirectness from one source (population, intervention, comparison, outcome); two levels if there was indirectness from more than one source; [5] imprecision: one level if the total sample size (sum of both groups) did not meet the optimal information size (OIS) criterion, defined as fewer than 400 participants; two levels when the CI around the effect estimate included meaningful effect and no effect; [6] publication bias: one level if publication bias was suspected. Quality of evidence will be classified into four categories: very low, low, moderate, and high. The assessment was conducted independently by two reviewers using the GRADEpro software (https://gradepro.org/).

### 2.8 Statistical Analysis

A random‐effects meta‐analysis was performed. Continuous outcomes were presented as standardized mean differences (SMD) with 95% confidence intervals (95% CI). Effect sizes were classified as minimal, small, medium, or large, corresponding to SMD values of < 0.2, 0.2–0.5, 0.5–0.8, and > 0.8, respectively [22]. Studies with multiple treatment groups were treated as independent studies. In instances where the control group sample was used more than once within the same forest plot, the sample size was halved. When more than one instrument was used to assess the same outcome (e.g., for pain, both SF‐36 and WOMAC), preference was given to the instrument specifically validated for assessing symptoms of the health condition under investigation, rather than a generic instrument applicable to any condition [23]. Assessment timepoints were categorized as short‐term (≤ 12 weeks), medium‐term (6 months), and long‐term (12 months) [24]. If multiple post‐intervention timepoints had been reported, the data closest to the timepoints of interest (12 weeks, 6 months, or 12 months) would have been selected.

Heterogeneity was evaluated through visual inspection of the forest plots and quantified using the *I* ^2^ statistic [15]. *I* ^2^ values were interpreted as follows: 0%–40% indicates minimal heterogeneity; 30%–60% reflects moderate heterogeneity; and 50%–90% indicates substantial heterogeneity, with significance set at *p* < 0.10. To examine the influence of individual studies on the pooled outcomes, a leave‐one‐out sensitivity analysis was applied. When sufficient data were available, subgroup analyses were planned according to participant age (adults vs. older adults).

Publication bias was assessed through inspection of funnel plots and Egger's test when ten or more studies were included for the same outcome. Statistical significance was defined as *p* < 0.05. All analyses were conducted using RevMan 5.4 software.

## 3 Results

### 3.1 Study Selection

**FIGURE 1:**
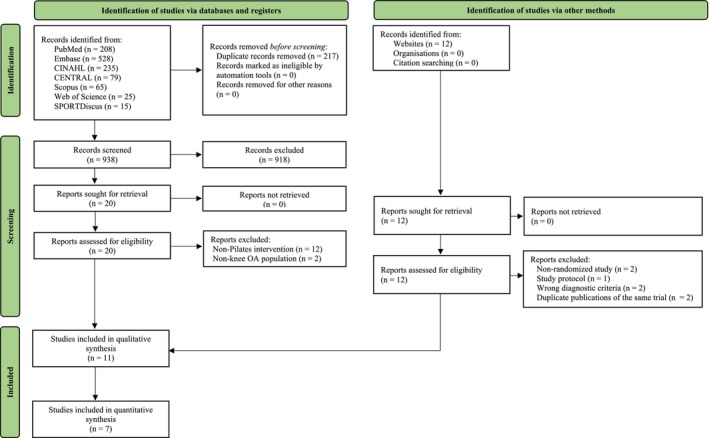
PRISMA flow diagram which included searches of databases, registers and other sources.

The literature search identified 1166 articles. The PRISMA flow diagram summarizes the results of the literature search (Figure 1). Eleven studies [11, 14, 25, 26, 27, 28, 29, 30, 31, 32, 33] were included in the qualitative analysis, of which seven studies [11, 14, 25, 28, 29, 32, 33] provided sufficient information for inclusion in the meta‐analysis. No subgroup analyses were conducted because none of the included studies provided outcome data stratified by age group.

### 3.2 Population and Study Characteristics

A total of 476 participants were included in the studies, with ages ranging from 43 to 68 years. The majority of participants were diagnosed with KOA according to the Kellgren and Lawrence criteria, ranging from levels 1–3, or by the American College of Rheumatology criteria. Eight of the included studies were two‐arm trials [25, 27, 28, 29, 30, 31, 32, 33], and three were three‐arm trials [11, 14, 26]. The studies were conducted in Brazil, Egypt, India, Iran, Nigeria, Pakistan, and Turkey. The publications of the studies ranged from 2013 to 2023. All studies are randomized clinical trials, with one of them being a conference abstract (Table 1).

**TABLE 1:** Characteristics of the included studies.

Study	Design	Sample size and profile	Diagnostic criteria	Pilates intervention	Comparison	Outcomes
Karimi 2021	RCT	*n* = 30 Country: Iran Setting: Outpatient Age (years): 61.4% ± 4.8% females: 100 BMI (kg/m^2^): NR Baseline pain: NR	Kellgren and Laurence	Pilates: seven exercises aimed at muscle strengthening, endurance, postural stability, proprioception and mobility 8 weeks (3×/week)	1. Suspension training: seven TRX suspension exercises targeting muscle strengthening, endurance, and stability 2. Control group: no intervention	Static balance Stork test (seconds; ↑ = better) Dynamic balance Y test (centimeters; ↑ = better) Knee range of motion Goniometry (degrees) Knee function WOMAC (0–100; ↑ = worse)
Rêgo 2023	RCT	*n* = 17 Country: Brazil Setting: Outpatient Age (years): 52.1% ± 8.9% females: 100 BMI (kg/m^2^): 30.5 ± 2.5 Baseline pain: 8.4 ± 2.6 (WOMAC)	Kellgren and Laurence (grade 2 or 3)	Mat Pilates: seven exercises divided into stretching, core exercises and relaxation. Exercises included movements such as leg raises, sidekicks, swimming, spine twists, and bridges 7 weeks (2×/week)	Control group: no intervention	Knee function WOMAC (0–100; ↑ = worse) Quality of life SF‐36 (0–100; ↑ = better)
Mazloum 2018	RCT	*n* = 41 Country: Iran Setting: NR Age (years): 52.1% ± 8.9% females: 32 BMI (kg/m^2^): NR Baseline pain: 10.4 ± 1.8 (Lequesne index)	American College of Rheumatology	Pilates: protocol based on pilates rehabilitation program after total hip and knee arthroplasty (Levene et al., 2007) 8 weeks (3×/week)	1. Conventional therapeutic exercise: isometric and concentric contractions aimed at strengthening specific muscles, while promoting stability and control 2. Control group: no intervention	Joint position sense Biodex 60° flexion (↓ error = better) Functional performance AFPT (time to complete walk, chair rise, stairs; ↓ = better) Pain and disability Lequesne index (0–24; ↑ = worse)
Saleem 2022	RCT	*n* = 40 Country: Pakistan Setting: NR Age (years): 56.6% ± 6.8% females: 100 BMI (kg/m^2^): 26.3 ± 4.2 Baseline pain: 8.0 ± 1.5 (VAS)	Kellgren and Laurence (grade 2 or 3)	Pilates: postural training, balance, breathing, and strength, focusing on quadriceps and gluteus strengthening, hip flexibility, and motor control, incorporating exercises such as bridging, squats, and specific stretches 8 weeks (3×/week)	Conventional therapeutic exercises: hot compresses, TENS, followed by isometric quadriceps strengthening and hamstring stretching exercises	Pain NPRS (0–10; ↑ = worse) Knee function WOMAC (0–100; ↑ = worse) Knee range of motion Goniometry (degrees)
Rabiei 2023	RCT	*n* = 54 Country: Iran Setting: Outpatient Age (years): 60.5% ± 5.6% females: 41 BMI (kg/m^2^): 29.5 ± 4.4 Baseline pain: 54.1 ± 13.2 (VAS 0–100)	American College of Rheumatology classification and Kellgren and Lawrence (grade 2 or 3)	Pilates: exercises were guided by six core principles: centering, control, precision, concentration, breath, and flow, ensuring mindful, coordinated, and fluid movements that emphasize spinal protection, core engagement, and full‐body integration 8 weeks (3×/week)	Pilates + pain neuroscience education: reframe negative beliefs about pain, reduce fear‐avoidance behaviors, and increase self‐efficacy by teaching participants about the mechanisms of pain using accessible explanations, visual aids, and recorded materials	Pain **WOMAC** (0–20; ↑ = worse) Physical function WOMAC (0–68; ↑ = worse) Pain catastrophizing PCS (0–52; ↑ = worse) Kinesiophobia TSK (17–68; ↑ = worse) Pain self‐efficacy PSEQ (0–60; ↑ = better) Functional performance TUG (seconds; ↑ = worse)
Akodu 2017	RCT	*n* = 33 Country: Nigeria Setting: Outpatient Age (years): 56.4% ± 11.6% females: 85 BMI (kg/m^2^): 29.7 ± 5.4 Baseline pain: 7.7 ± 1.2 (VAS)	Kellgren and Laurence	Pilates: exercises + TENS 8 weeks (2×/week)	1. Isometric exercise + TENS 2. Usual care: diet education, losing weigth, knee care education, joint protection measures and TENS	Pain VAS (0–10; ↑ = worse) Knee range of motion Goniometry (degrees) Knee function WOMAC (0–100; ↑ = worse)
Bakk 2023	RCT	*n* = 30 Country: Egypt Setting: Outpatient Age (years): 51.9% ± 3.7% females: 0 BMI (kg/m^2^): < 30 Baseline pain: 8.3 ± 0.8 (VAS)	Diagnosed by an orthopedist (unspecified criterion)	Pilates: Pilates‐based exercises targeting abdominal muscles, core stability, spinal muscle stretching and postural control + the same procedures as the usual care group 8 weeks (3×/week)	Usual care: traditional exercise programme, infrared therapy, and US application	Pain VAS (0–10; ↑ = worse) Knee range of motion Goniometry (degrees) Knee function WOMAC (0–100; ↑ = worse)
Meenakshi 2021	RCT	*n* = 68 Country: India Setting: Outpatient Age (years): 49% females: NR BMI (kg/m^2^): NR Baseline pain: 5.9 ± 1.2 (VAS)	American College of Rheumatology classification and Kellgren and Lawrence (grade 1 and 2)	Pilates: exercises focused on core strengthening, movement control, body alignment, and functional mobility, including the hundred, one leg stretch, double leg stretch, clams, side leg kick, and one leg circle 6 weeks (3×/week)	Closed kinematic chain exercises: balance training on hard and soft surfaces, retro walking, stair climbing, heel raises, single‐leg stance with dynamic leaning, and sit‐to‐stand transitions	Pain VAS (0–10; ↑ = worse) Knee flexor isometric strength Hand held dynamometer (kg) Knee function WOMAC (0–100; ↑ = worse)
Meenakshi 2021a	RCT	*n* = 64 Country: India Setting: Outpatient Age (years): 53% females: NR BMI (kg/m^2^): NR Baseline pain: 6.0 ± 1.6 (VAS)	American College of Rheumatology classification and Kellgren and Lawrence	Pilates: exercises focused on core strengthening, movement control, body alignment, and functional mobility, including the hundred, one leg stretch, double leg stretch, clams, side leg kick, and one leg circle (same of Meenakshi 2021) 8 weeks (3×/week)	Neuromuscular exercises: balance, coordination, and dynamic postural control through exercises such as tandem walking, side and crossover stepping, modified grapevine, and variations of forward and backward marching	Pain VAS (0–10; ↑ = worse) Knee function WOMAC (0–100; ↑ = worse)
Kisacik 2016	RCT (Conference abstract)	*n* = 39 Country: Turquia Setting: NR Age (years): 55.9% ± 5.5% females: NR BMI (kg/m^2^): NR Baseline pain: NR	Kellgren and Lawrence (grade 1 and 2)	Clinical Pilates exercise 10 weeks (3×/week)	Control group: no intervention	Kinestesia and position sense PMS‐1000; degrees (↓ error = better)
Rajinder 2016	RCT	*n* = 60 Country: India Setting: NR Age (years): 55.7% ± 3.2% females: NR BMI (kg/m^2^): 26.9 ± 1.7 Baseline pain: 5.4 ± 0.9 (VAS)	Kellgren and Lawrence (grade 1 and 2)	Pilates: increasing complexity and repetitions weekly with exercises such as hundreds, adding one‐leg stretch, double‐leg stretch, clams, one‐leg kick, side kick, and one‐leg circle, with repetitions increasing from 5 to 10 6 weeks (3×/week)	Proprioceptive exercises: one‐leg balances, leg swings, toe and heel walking, and cross‐body swings, progressing to advanced one‐leg balances, maximum swings, squats, runner's poses, blind balances, bicycle swings, and partial squats	Pain NPRS (0–10; ↑ = worse) Knee function WOMAC (0–100; ↑ = worse) Joint position sense Goniometer 30° flexion (↓ error = better)

*Note:* Data are presented as mean ± standard deviation or as percentages. Abbreviations: AFPT, aggregate functional performance time; KOA, knee osteoarthritis; NPRS, numeric pain rating scale; NR, not reported; PCS, pain catastrophizing scale; PSEQ, pain self‐efficacy questionnaire; RCT, randomized controlled trial; SF‐36, Short‐Form 36; TENS, transcutaneuous electrical nerve stimulation; TRX, total resistance exercise; TSK, tampa scale for kinesiophobia; TUG, timed up and go; US, ultrasound; VAS, visual analog scale; WOMAC, Western Ontario & McMaster University osteoarthritis index.

### 3.3 Summary of the Pilates Interventions

The intervention period and frequency were substantially consistent across most studies, ranging from 6 to 10 weeks (short‐term) with sessions conducted 2–3 times per week. Protocols followed the core principles of Pilates (centering, control, precision, concentration, breathing, and flow), incorporating a progressive structure and emphasizing core strengthening, flexibility, postural control, and breathing. While specific exercises and progression schemes varied, common movements included “the hundred”, “single leg stretch”, “double leg stretch”, “clam”, and “shoulder bridge”. Some studies also introduced more advanced or combined modalities (e.g., clinical Pilates, Pilates plus TENS), with adaptations based on participant capacity. For further details of the interventions, see Supporting Information S2.

### 3.4 Adverse Events

Only two studies provided data on adverse events. Rêgo et al. (2023) stated that no participant experienced adverse effects related to the intervention, such as pain or musculoskeletal discomfort, during the treatment or between sessions [32]. Rabiei et al. (2023) reported no serious adverse events in the pain neuroscience education followed by Pilates exercises group or in the Pilates exercises group [30].

### 3.5 Meta‐Analysis

#### 3.5.1 Effect of Pilates on Pain

**FIGURE 2:**
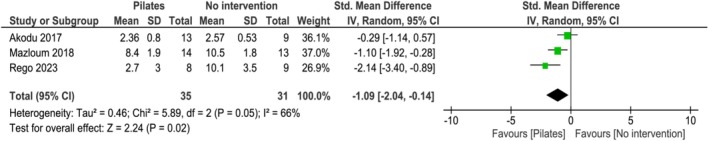
Forest plot of comparison: Pilates versus no intervention; outcome: Pain (short‐term). CI, confidence interval; SD, standard deviation; STD, standardized.

**FIGURE 3:**
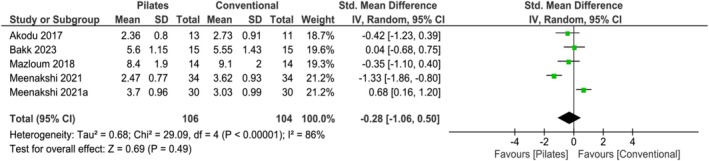
Forest plot of comparison: Pilates versus conventional exercise; outcome: Pain (short‐term). CI, confidence interval; SD, standard deviation; STD, standardized.

Based on 3 studies [11, 14, 32], Pilates exercises reduced pain compared with no intervention in the short‐term (SMD −1.09; 95% CI −2.04 to −0.14; *I* ^2^ = 66%; *p* = 0.02; 3 studies; *n* = 66; low‐quality evidence) (Figure 2). Based on 5 studies [11, 14, 25, 28, 29], Pilates exercises did not reduce pain compared with conventional exercises in the short‐term (SMD −0.28; 95% CI −1.06 to 0.50; *I* ^2^ = 86%; *p* = 0.49; 5 studies; *n* = 210; very low‐quality evidence) (Figure 3).

#### 3.5.2 Effect of Pilates on Knee Health

**FIGURE 4:**
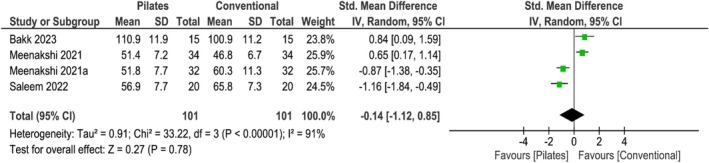
Forest plot of comparison: Pilates versus conventional exercise; outcome: Knee health (short‐term). CI, confidence interval; SD, standard deviation; STD, standardized.

Based on 4 studies [25, 28, 29, 33], Pilates exercises did not increase knee health assessed with the Western Ontario and McMaster Universities Osteoarthritis Index (WOMAC) compared with conventional exercises in the short‐term (SMD −0.14; 95% CI −1.12 to 0.85; *I* ^2^ = 91%; *p* = 0.78; 4 studies; *n* = 202; very low‐quality evidence) (Figure 4).

#### 3.5.3 Effect of Pilates on Knee Range of Motion

Based on 2 studies [25, 33], Pilates exercises increased knee range of motion compared with conventional exercises in the short‐term (SMD 1.07; 95% CI 0.56 to 1.57; *I* ^2^ = 0%; *p* = 0.0001; 2 studies; *n* = 70; low‐quality evidence) (Supporting Information S3).

### 3.6 Descriptive Synthesis

#### 3.6.1 Balance and Proprioception

Three trials [11, 26, 27], comprising 110 participants, investigated the effects of Pilates on balance and proprioception. These studies could not be included in the meta‐analysis due to heterogeneity in outcome measures and lack of standardized effect size reporting. Two of these studies demonstrated low methodological quality and were classified as having a high risk of bias [26, 27], while one study was classified with some concerns [11].

The study by Karimi et al. (2021) analyzed the effects of suspension training and Pilates on the balance of patients with KOA [26]. A significant increase in dynamic balance was observed in the anterior (*p* ≤ 0.04), posteromedial (*p* ≤ 0.05), and posterolateral (*p* ≤ 0.04) directions in both experimental groups, with no improvement in the control group (*p* ≥ 0.09). Static balance also improved significantly in the intervention groups (*p* = 0.001) while the control group presented no difference (*p* = 0.50).

Mazloum et al. (2018) assessed Target Angle Reproduction Error (TARE) to measure knee proprioception, comparing the effects of Pilates and conventional therapeutic exercises [11]. A significant improvement in proprioceptive accuracy was found in both experimental groups, with a significant reduction in target angle reproduction error (*p* < 0.001). However, there was no statistically significant difference between the intervention groups (*p* = 0.727).

The study by Kisacik et al. (2016) found a significant improvement in kinesthetic perception (the ability to perceive body movement and position in space) in the exercise group (*p* < 0.05) [27]. However, no significant improvement was observed in position sense (the ability to perceive the position of a joint without visual aid) in the same group (*p* > 0.05). Additionally, the control group, which did not perform the exercises, showed no changes in kinesthetic and position sense values before and after treatment.

#### 3.6.2 Quality of Life

One trial [32], comprising 41 participants, investigated the effects of Pilates on quality of life. Because this was the only study evaluating this outcome, it could not be included in the meta‐analysis. This study demonstrated low methodological quality and was classified as having a high risk of bias due to the lack of blinding of outcome assessors.

The study conducted by Rêgo et al. (2023) evaluated the quality of life of older women with KOA using the SF‐36 questionnaire [32]. The experimental group, which performed mat Pilates, showed a significant improvement in functional capacity (33.12 ± 22.03 to 69.37 ± 22.43; *p* < 0.05) and in the pain domain (39.50 ± 12.89 to 71.75 ± 18.66; *p* < 0.05). Furthermore, overall health status also improved in the Pilates group (59.44 ± 18.07 to 82.75 ± 12.03; *p* < 0.05), whereas no significant changes were observed in the control group, which did not receive any physical intervention or therapeutic guidance.

### 3.7 Risk of Bias

**FIGURE 5:**
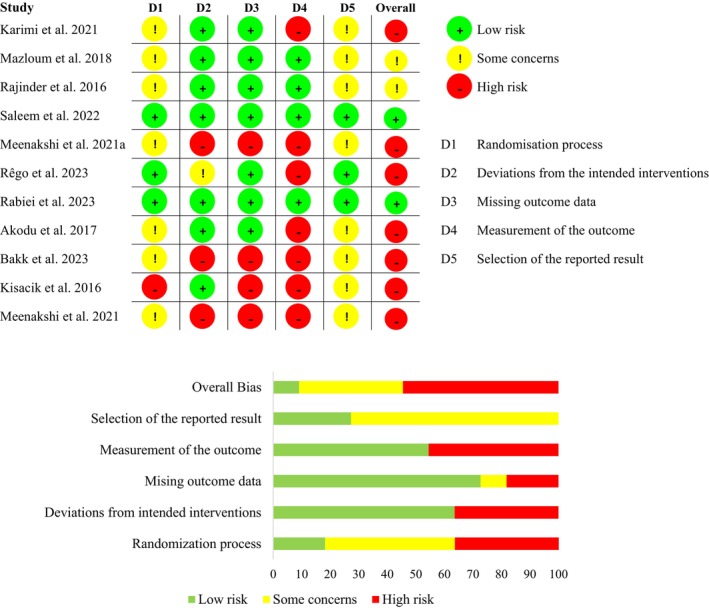
Summary plots of the risk of bias.

Many studies exhibited a high risk of bias, particularly in the domain of outcome measurement, mainly due to the lack of blinding of outcome assessors when evaluating subjective outcomes such as pain and function. On the other hand, in the majority of studies, greater methodological quality was observed in the domains of missing outcome data and deviations from intended interventions (Figure 5).

### 3.8 Quality of Evidence

Although all analyzed outcomes derived from randomized clinical trials, the quality of the evidence was downgraded due to methodological limitations. Most studies exhibited a high risk of bias, leading to a reduction in confidence in the results. Additionally, two comparisons showed high inconsistency among the included studies, resulting in a further downgrading of the quality of evidence. However, for the domain of indirectness, no limitations were identified that would justify an additional downgrade. Conversely, all the studies had sample sizes below the Optimal Information Size (OIS) and possessed wide confidence intervals, leading to a downgrading in the quality of evidence due to imprecision. Consequently, the overall confidence in the findings ranged from low to very low (Supporting Information S4).

## 4 Discussion

The results of this systematic review indicate that Pilates exercises may provide benefits for individuals with KOA. The analysis of the included studies revealed that Pilates might help reduce pain compared with no intervention and may improve knee range of motion compared to conventional exercises, both having a large effect size. Additionally, the evidence supporting a positive impact of Pilates practice on proprioception and dynamic balance is very uncertain. No significant differences were observed in knee health improvement, as assessed according to the WOMAC scale, when compared with other conventional exercise methods; however, the evidence is also very uncertain. These findings suggest that Pilates may be a viable alternative for the rehabilitation of patients with KOA, particularly in terms of pain reduction and motor control improvement. Most of the included studies involved middle‐aged adults, a participant profile that should be taken into account when interpreting the results, given the limited investigation of older adults. Although these results are promising, the quality of the evidence ranged from very low to low, indicating that the findings should be interpreted with caution.

The present systematic review highlights the benefits of Pilates as an intervention for KOA while its effectiveness is comparable to that of other conventional exercises, although this evidence is very uncertain. Raposo et al. (2021) reported that both aerobic and strengthening exercises effectively reduce pain and improve function in individuals with KOA [34]. Similarly, the findings of the present review indicate that Pilates may reduce pain when compared with no intervention. Moreover, the results suggest, with a high degree of uncertainty in the evidence, that its effectiveness is similar to that of conventional exercise protocols. Likewise, Denham‐Jones et al. (2022) concluded that Pilates is as effective as other exercises in managing pain and functional impairment in older adults with chronic musculoskeletal conditions, without evidence of significant differences between approaches [35]. In comparison, the present review focused specifically on knee osteoarthritis, included additional outcomes such as range of motion, balance, proprioception, and adverse events, and incorporated several recent randomized controlled trials. Furthermore, we conducted quantitative meta‐analyses and assessed the certainty of the evidence using GRADE, providing a more robust and up‐to‐date synthesis of the available literature.

Additionally, our review identified potential improvements in knee range of motion and both dynamic and static balance, reinforcing previous findings that Pilates may enhance proprioception and postural stability. In contrast to Denham‐Jones et al. (2022), who reported broad quality‐of‐life benefits across various musculoskeletal conditions [35], our synthesis highlighted more specific gains in functional capacity, pain, and overall health status measured by the SF‐36 in elderly women with KOA, although this evidence remains very uncertain. Taken together, these findings support Pilates as a potential therapeutic modality for KOA, while also suggesting that its benefits relative to other exercise interventions may depend on the outcomes assessed and the characteristics of the target population.

For clinical practice, the Pilates method may be a strategy for the rehabilitation of patients with KOA, particularly in reducing pain and improving range of motion, although the evidence regarding pain compared with conventional treatment is very uncertain. Although Pilates has shown effects on knee health comparable to conventional exercises, as well as potential additional benefits in proprioception and dynamic balance, the quality of evidence for these outcomes is very low. Therefore, while its incorporation into clinical practice may be a viable alternative for patients with deficits in these areas, these findings should be interpreted with caution. Furthermore, the progressive and adaptable nature of Pilates exercises allows for personalization in keeping with the condition and progression of the patient, which may enhance adherence to treatment. However, due to the heterogeneity of intervention protocols and variability in outcomes, healthcare professionals are encouraged to customize the prescription of Pilates based on a comprehensive physical‐functional assessment and integrate it with other established therapeutic approaches for KOA management.

Future studies on the impact of the Pilates method on KOA should adopt more robust methodological designs, with larger sample sizes and longer follow‐up periods to assess the sustainability of intervention effects, identify potential adverse events, and evaluate long‐term adherence. Additionally, standardization of intervention protocols is recommended, including a detailed description of the exercises performed, frequency, and progressive intensity, in order to enhance replicability. Further research should also explore the effects of Pilates in subgroups of adults and older adults, incorporate complementary outcomes such as knee extensor and flexor strength, quality of life, and functional capacity measures, such as gait speed and sit‐to‐stand performance, to better elucidate the action mechanisms of the method and identify patient profiles that may derive the greatest benefit from this intervention.

This systematic review is not without its limitations. Although the included studies adequately described the prescribed exercises, most did not specify the target intensity, the criteria for its definition, or whether progression occurred throughout the sessions, all of which may compromise the accuracy of the results. Additionally, some outcomes, such as balance, proprioception, and quality of life, could not be pooled for analysis using statistical methods due to the scarcity of investigations into these variables and the heterogeneity among the studies. Furthermore, most investigations assessed the effects of Pilates only in the short‐to‐medium term, making it difficult to extrapolate the findings to longer periods. For outcomes such as pain and knee health, the high statistical heterogeneity observed indicates considerable variability among the pooled studies. Finally, although most risk of bias assessment domains were rated as having a low risk, the majority of the studies were assessed as having a high overall risk of bias, indicating that the results of this review should be interpreted with caution.

This systematic review presents several strengths. The study was conducted following internationally recognized guidelines, such as the Cochrane Handbook and PRISMA criteria, ensuring transparency, methodological rigor, and reproducibility in the synthesis of evidence. Additionally, the search strategy was comprehensive, including multiple relevant databases without restrictions as to language or publication date, thereby minimizing the risk of publication bias. The inclusion in the study of exclusively randomized clinical trials enhances the quality of evidence and reduces potential biases associated with other study designs, for example, non‐randomized studies. Furthermore, the quality of evidence was assessed using the GRADE tool, permitting an evaluation of whether future studies may impact the findings of this review.

## 5 Conclusion

The Pilates method may be an effective alternative for the rehabilitation of patients with KOA, particularly in reducing pain when compared with no intervention. However, Pilates did not demonstrate superiority over conventional exercises in reducing pain or improving knee health as assessed by the WOMAC. On the other hand, the method may enhance knee range of motion compared with conventional exercises and may also provide benefits in proprioception and dynamic balance, although the evidence for these outcomes is very uncertain. Furthermore, Pilates exercises had a positive impact on quality of life, particularly in functional capacity, pain perception, and the overall health of older women with KOA, with the evidence for this outcome also being very uncertain. The heterogeneity among the included studies, as well as the predominance of short‐term investigations, limits the generalizability of the findings to medium‐ and long‐term interventions. The very low to low quality of the evidence indicates that the results should be interpreted with caution and that new randomized clinical trials may modify the findings of this review.

## Author Contributions

T.M.D.O.: conceptualization, methodology, software, formal analysis, writing of the original draft, and visualization; D.C.F.: conceptualization, writing – review and editing, supervision, project administration, and funding acquisition; J.E.F.: writing of the original draft, visualization, and formal analysis; RQC: writing of the original draft, and visualization; F.J.S.G.: methodology, writing of the original draft, and visualization; L.F.P.P.: methodology, writing of the original draft, and visualization; D.S.F.: resources, writing – review and editing, supervision, and visualization; C.M.: conceptualization, methodology, resources, writing – review and editing, supervision, project administration, and funding acquisition.

## Conflicts of Interest

The authors declare no conflicts of interest.

## Supporting information


**Appendix S1:** apl70434‐sup‐0001‐AppendixS1.docx.

**Appendix S2:** apl70434‐sup‐0002‐AppendixS2.docx.

**Appendix S3:** apl70434‐sup‐0003‐AppendixS3.docx.

**Appendix S4:** apl70434‐sup‐0004‐AppendixS4.docx.

## Data Availability

This study is a systematic review and meta‐analysis. All data analyzed are included in the published articles cited in the reference list. Additional data extraction sheets and analysis files are available from the corresponding author upon reasonable request.
