# Supplementary material for: Effect of Pilates Exercise on Health‐Related Outcomes in Patients With Knee Osteoarthritis: A Systematic Review and Meta‐Analysis
Source: Int J Rheum Dis. 2025 Oct 9;28(10):e70434. doi: 10.1111/1756-185x.70434 (PMC12509171; doi:10.1111/1756-185x.70434)
Supplement: Supplementary file 1 — Appendix S1: apl70434‐sup‐0001‐AppendixS1.docx. [file APL-28-e70434-s001.docx]

Supplementary Material 1. Search strategy for each database.

**PubMed**

1. pilates OR clinical pilates OR pilates training OR pilates method OR pilates exercise OR exercise movement techniques[MeSH Terms]

2. knee osteoarthritis[MeSH Terms] OR knee joint[MeSH Terms] OR knee[MeSH Terms] OR knee osteoarthrosis OR knee AO OR gonarthrosis OR degenerative arthritis

3. randomized controlled trial OR clinical trial OR randomized OR randomised OR trial OR rct OR randomized clinical trial OR non-randomized OR non-randomized stud* OR quase-experimental

4. #1 AND #2 AND #3

**Embase**

‘Pilates’/exp OR ‘Clinical Pilates’:ab,ti OR ‘Pilates Training’:ab,ti OR ‘Pilates Method’:ab,ti OR ‘Pilates Exercise’:ab,ti OR ‘Kinesiotherapy’/exp

‘Knee Osteoarthritis’:ti,ab OR ‘Knee Osteoarthrosis’:ti,ab OR ‘Knee OA’:ti,ab OR ‘Gonarthrosis’:ti,ab

‘Randomized Controlled Trial’:ti OR ‘Clinical Trial’:ti OR ‘Randomized’:ti OR ‘Randomised’:ti OR ‘Trial’:ti OR ‘RCT’:ti OR ‘Randomized Clinical Trial’:ti OR ‘Controlled clinical trial’:ti OR ‘Non-Randomized’:ti OR ‘Non-Randomized Study’:ti OR ‘Quasi-Experimental Study’:ti

**Cochrane library**

1. MeSH descriptor: [Exercise Movement Techniques] explode all trees

2. pilates

3. clinical pilates

4. pilates training

5. pilates method

6. pilates exercise

7. #1 OR #2 OR #3 OR #4 OR #5 OR #6

8. MeSH descriptor: [Osteoarthritis, Knee] explode all trees

9. MeSH descriptor: [Knee Joint] explode all trees

10. MeSH descriptor: [Knee] explode all trees

11. knee osteoarthrosis

12. knee OA

13. gonarthrosis

14. #8 OR #9 OR #10 OR #11 OR #12 OR #13

15. MeSH descriptor: [Randomized Controlled Trial] explode all trees

16. MeSH descriptor: [Clinical Trial] explode all trees

17. trial

18. rct

19. “randomized clinical trial”

20. #15 OR #16 OR #17 OR #18 OR #19

21. #7 AND #14 AND #20

**CINAHL**

TX (Pilates OR Clinical Pilates OR Pilates Training OR Pilates Method OR Pilates Exercise OR Exercise Movement Techniques) AND TX (Knee Osteoarthritis OR Knee Joint OR Knee OR Knee Osteoarthrosis OR Knee OA OR Gonarthrosis OR Degenerative Arthritis) AND TI (Randomized Controlled Trial OR Clinical Trial OR Randomized OR Randomised OR Trial OR RCT OR Randomized Clinical Trial OR Non-Randomized OR Non-Randomized Stud* OR Quasi-Experimental)

**Scopus**

1. “pilates” OR “clinical pilates” OR “pilates training” OR “pilates method” OR “pilates exercise” OR “exercise movement technique”

2. “knee osteoarthritis” OR “knee joint” OR “knee” OR “knee osteoarthrosis” OR “knee OA” OR “gonarthrosis” OR “degenerative arthritis”

3. “randomized controlled trial” OR “clinical trial” OR “randomized” OR “randomised” OR “trial” OR “rct” OR “randomized clinical trial” OR “non-randomized” OR “non-randomized study” OR “quase-experimental”

4. #1 AND #2 AND #3

**Web of Science**

1. “pilates” OR “clinical pilates” OR “pilates training” OR “pilates method” OR “pilates exercise” OR “exercise movement technique”

2. “knee osteoarthritis” OR “knee joint” OR “knee” OR “knee osteoarthrosis” OR “knee OA” OR “gonarthrosis” OR “degenerative arthritis”

3. “randomized controlled trial” OR “clinical trial” OR “randomized” OR “randomised” OR “trial” OR “rct” OR “randomized clinical trial” OR “non-randomized” OR “non-randomized study” OR “quase-experimental”

4. #1 AND #2 AND #3

**SPORTDiscus**

1. “pilates” OR “clinical pilates” OR “pilates training” OR “pilates method” OR “pilates exercise” OR “exercise movement technique”

2. “knee osteoarthritis” OR “knee joint” OR “knee” OR “knee osteoarthrosis” OR “knee OA” OR “gonarthrosis” OR “degenerative arthritis”

3. “randomized controlled trial” OR “clinical trial” OR “randomized” OR “randomised” OR “trial” OR “rct” OR “randomized clinical trial” OR “non-randomized” OR “non-randomized study” OR “quase-experimental”

4. #1 AND #2 AND #3
