# Supplementary material for: Effect of Pilates Exercise on Health‐Related Outcomes in Patients With Knee Osteoarthritis: A Systematic Review and Meta‐Analysis
Source: Int J Rheum Dis. 2025 Oct 9;28(10):e70434. doi: 10.1111/1756-185x.70434 (PMC12509171; doi:10.1111/1756-185x.70434)
Supplement: Supplementary file 2 — Appendix S2: apl70434‐sup‐0002‐AppendixS2.docx. [file APL-28-e70434-s003.docx]

Supplementary Material 2. Pilates exercise characteristics.

The intervention period and frequency were substantially consistent across most studies, ranging from 6 to 10 weeks with sessions conducted 2 to 3 times per week.

The Pilates exercise program applied in the five studies (1-5) were very similar, based on the principles of the Pilates method (centering, control, precision, concentration, breathing, and flow) (6) and were conducted over a period of 6 to 8 weeks, with 60-minute sessions performed three times per week. The protocols followed a well-defined weekly progression, starting with the introduction of basic movements during the first few weeks, which included a lower number of repetitions, generally five, and shorter durations of 20 to 30 seconds. In the intermediate weeks, new exercises were added along with a gradual increase in the number of repetitions from six to eight or an extended period of execution. Each session was structured in three phases: warm-up (10 minutes), main exercises (40 minutes), and relaxation (10 minutes). The exercises included movements such as the "the hundred", "single leg stretch", "double leg stretch", "clam", and "shoulder bridge". More advanced exercises, such as "scissors" and the "side kick series", were progressively introduced.

Two studies (7, 8) implemented similar interventions over a period of 7 to 8 weeks, with 60-minute sessions conducted 2 to 3 times per week. Each session included 10 minutes of warm-up, 40 minutes of main exercises, and 10 minutes of relaxation, with weekly adaptations and progressions. All the protocols emphasized core strengthening, postural control, flexibility, and breathing, featuring exercises such as "the hundred", "single leg stretch", "double leg stretch", "clam", and "shoulder bridge." Rêgo et al., 2023 (7), incorporated dynamic exercises like "side kicks", "swimming", and "spine twist", while Saleem et al., 2022 (8), followed a more detailed progression scheme, gradually adding exercises like "single leg kick" and "side kick."

Two studies (9, 10) prescribed protocols with a duration of 8 weeks, with sessions conducted 2 to 3 times per week. The protocol employed by of Bakk et al. (2023) (10) is more detailed, encompassing specific categories of exercises such as warm-up, abdominal strengthening, spinal stretching, and advanced series like the "swan series" and "side kick series," with an emphasis on controlled breathing and adjustment of repetitions in accordance with the respective abilities of the participants. In contrast, the study by Akodu et al. (2017) (9) combines Pilates with transcutaneous electrical nerve stimulation (TENS), primarily focusing on muscle strengthening and pain relief, with a less specific description of the Pilates exercises. Both studies include exercises such as “roll up”, "single-leg circle", and "shoulder bridge", but Bakk et al. (2023) (10) incorporates a progression to advanced movements such as "swan-neck roll”, "side kick front/back”, and "open leg rocker".

Karimi et al. (2021) (11) investigated a protocol lasting 8 weeks, with sessions conducted three times a week. The program included postural exercises, balance, breathing, strength, and endurance, with progression based on increasing repetitions. One of the main differentiating factors of this protocol was the inclusion of squats, hip abduction exercises, and plantar flexor exercises, with intensity adjusted based on heart rate. In contrast, Kisacik et al. (2016) (12) conducted a study with a longer intervention duration of 10 weeks, with sessions also held three times a week. However, information regarding the structure of the program is limited, as the study only mentions the use of clinical Pilates without detailing specific exercises or session organization.

**References**

1. Mazloum V, Rabiei P, Rahnama N, Sabzehparvar E. The comparison of the effectiveness of conventional therapeutic exercises and Pilates on pain and function in patients with knee osteoarthritis. Complement Ther Clin Pract 2018;31:343-8.

2. Meenakshi C, Apparao P, Chaturvedi A, Mounika R, Chintada D. Comparison of Pilates exercises and closed kinematic chain exercises on pain, muscle strength and functional performance in subjects with knee osteoarthritis. J Physiother Res 2021;5:1.

3. Meenakshi C, Apparao P, Chintada D, Geethamounika R, Prakash P. Effectiveness of neuromuscular exercises and Pilates exercises on pain and function in subjects with chronic knee osteoarthritis. Eur J Pharm Med Res 2021;8:359-68.

4. Rabiei P, Sheikhi B, Letafatkar A. Examining the influence of pain neuroscience education followed by a Pilates exercises program in individuals with knee osteoarthritis: a pilot randomized controlled trial. Arthritis Res Ther 2023;25:94.

5. Rajinder K, Harneet K. Comparison of Pilates exercises and proprioceptive exercises on joint position sense in people with knee osteoarthritis. Int J Sci Res 2016;5:907-11.

6. Wells C, Kolt GS, Bialocerkowski A. Defining Pilates exercise: a systematic review. Complement Ther Med 2012;20:253-62.

7. Rêgo TAM, Ferreira APL, Villela DW, Shirahige L, Xavier AB, Braz RRS, et al. Effects of mat Pilates on older adult women with knee osteoarthritis: a randomized controlled trial. J Bodyw Mov Ther 2023;33:136-41.

8. Saleem N, Zahid S, Mahmood T, Ahmed N, Maqsood U, Chaudhary MA. Effect of Pilates based exercises on symptomatic knee osteoarthritis: a randomized controlled trial. J Pak Med Assoc 2022;72:8-12.

9. Akodu A, Fapojuwo O, Quadri AA. Comparative effects of Pilates and isometric exercises on pain, functional disability and range of motion in patients with knee osteoarthritis. Res J Health Sci 2017;5:94.

10. Bakk A, Ahmed E. The effect of Pilates exercises on knee osteoarthritis. Egypt J Phys Ther 2023;14:35-41.

11. Karimi N, Dehkordi KJ, Rizi RM. Effects of Pilates training vs. suspension training on quality of life in women with knee osteoarthritis: a randomized controlled trial. J Bodyw Mov Ther 2021;27:737-45.

12. Kisacik P, Oksuz S, Arın G, Akdogan A, Dogan O, Karabulut E, et al. FRI0637-HPR The effects of clinical Pilates exercises on kinestesia and position sense in patients with osteoarthritis of the knee. Ann Rheum Dis 2016. p. 1284.2-.
