# Supplementary material for: Effect of Pilates Exercise on Health‐Related Outcomes in Patients With Knee Osteoarthritis: A Systematic Review and Meta‐Analysis
Source: Int J Rheum Dis. 2025 Oct 9;28(10):e70434. doi: 10.1111/1756-185x.70434 (PMC12509171; doi:10.1111/1756-185x.70434)
Supplement: Supplementary file 3 — Appendix S3: apl70434‐sup‐0003‐AppendixS3.docx. [file APL-28-e70434-s004.docx]

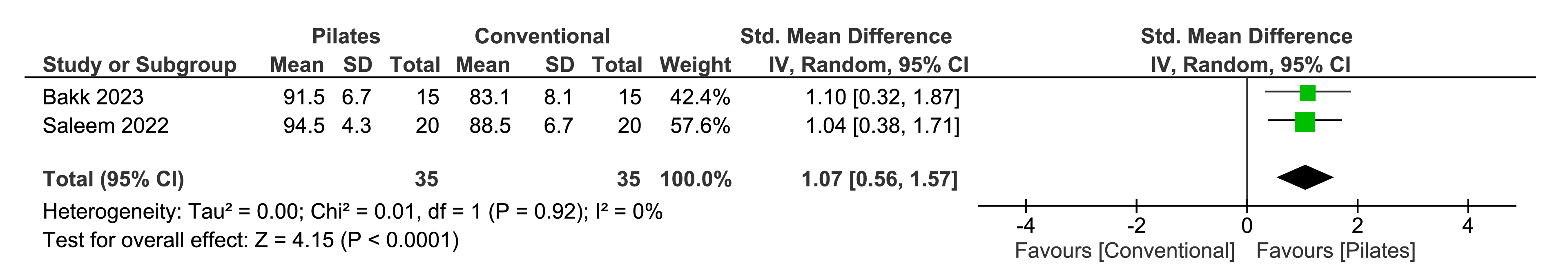


Supplementary Material 3. Forest plot of comparison: Pilates versus conventional exercise; outcome: range of motion (short-term). CI confidence interval, SD standard deviation, STD standardized.
