# Supplementary material for: Effect of Pilates Exercise on Health‐Related Outcomes in Patients With Knee Osteoarthritis: A Systematic Review and Meta‐Analysis
Source: Int J Rheum Dis. 2025 Oct 9;28(10):e70434. doi: 10.1111/1756-185x.70434 (PMC12509171; doi:10.1111/1756-185x.70434)
Supplement: Supplementary file 4 — Appendix S4: apl70434‐sup‐0004‐AppendixS4.docx. [file APL-28-e70434-s002.docx]

|  |  | **Certainty assessment** | | | | | | |  | **Nº of patients** | |  | **Effect** |  |  |  |
| --- | --- | --- | --- | --- | --- | --- | --- | --- | --- | --- | --- | --- | --- | --- | --- | --- |
| Outcome | Comparison | Nº of studies | Study design | Risk of bias | Inconsistency | Indirectness | Imprecision | Publication bias |  | Pilates | Control |  | Absolute (95% CI) |  | Certainty | Importance |
| Pain | Pilates x NI | 3 | RCT | serious^a^ | not serious | not serious | serious^c^ | N/A |  | 35 | 31 |  | SMD -1.09 (-2.04 - 0.14) |  | ⨁⨁◯◯ Low | Critical |
| Pain | Pilates x CE | 5 | RCT | serious^a^ | serious^b^ | not serious | serious^c^ | N/A |  | 106 | 104 |  | SMD 0.28 (-1.06 - 0.50) |  | ⨁◯◯◯ Very low | Critical |
| Knee health | Pilates x CE | 4 | RCT | serious^a^ | serious^b^ | not serious | serious^c^ | N/A |  | 101 | 101 |  | SMD -0.14 (-1.12 - 0.85) |  | ⨁◯◯◯ Very low | Critical |
| ROM | Pilates x CE | 2 | RCT | serious^a^ | not serious | not serious | serious^c^ | N/A |  | 35 | 35 |  | SMD 1.07 (0.56 – 1.57) |  | ⨁⨁◯◯ Low | Critical |

Supplementary Material 4. GRADE assessment summary.

Abbreviations: ROM range of motion, NI non-intervention, CE conventional exercise, RCT randomized controlled trial, N/A not applicable, SMD standardized mean difference

a. Risk of bias ranged from moderate to high according to RoB 2

b. Lack of overlap of confidence intervals between studies in the forest plot

c. The sample size is below the optimal information size (n=400)
